# Influence of Annealing Time on Microstructure and Mechanical Properties of Al-14.5Si Alloy Prepared by Super-Gravity Solidification and Cold-Rolling

**DOI:** 10.3390/ma15165475

**Published:** 2022-08-09

**Authors:** Zhanghua Gan, Qian Ni, Yuanyuan Huang, Yin Su, Yuehui Lu, Chuandong Wu, Jing Liu

**Affiliations:** 1 State Key Laboratory of Refractories and Metallurgy, Wuhan University of Science and Technology, Wuhan 430081, China; 2 Hubei Engineering Technology Research Center of Marine Materials and Service Safety, Wuhan 430081, China

**Keywords:** aluminum alloys, mechanical properties, super-gravity solidification, cold-rolling

## Abstract

In this paper, super-gravity solidification and cold-rolling were utilized to obtain Al-14.5Si alloys. The influence of annealing time on microstructure and mechanical properties of Al-14.5Si alloys was investigated. Our results indicated that high elongation was achieved by super-gravity solidification due to the submicron eutectic Si, making it possible to undertake the conventional cold-rolling. The yield strength (~214 ± 11 MPa) was significantly enhanced (~68.5%) after cold-rolling mainly due to high dislocation density. The coarsening of eutectic Si could be observed during annealing, which resulted in a decrease in yield strength. The elimination of internal stress and lattice distortion during annealing led to a decrease in micro-cracks/voids beneath the fracture surface during tensile testing, which in turn enhanced the elongation.

## 1. Introduction

Al-Si eutectic alloy, as an ideal wear-resistant and heat-resistant material, has broad application prospects in aerospace, automobile, railway, and electronic communication industries, probably due to its low density, good wear/corrosion resistance, excellent casting performance, low coefficient of thermal expansion [1,2,3,4]. Typically, the formation and propagation of cracks preferred to occur in the vicinity of needle-like eutectic Si under external force due to the stress concentration, which resulted in low strength, limited ductility and poor machinability [5,6,7]. Certain attempts were utilized to enhance the mechanical properties of the as-casted Al-Si alloys by the modification of brittle eutectic Si with a needle-like morphology and uneven distribution [8,9,10].

The refinement of eutectic Si could be achieved by chemical metamorphism (P, Na, Sr, La, Sb and composite modifiers) [11] and processing regulation (rapid solidification [12], melt pretreatment, low temperature casting, high pressure crystallization, ultrasonic vibration [13] and super-gravity solidification [14], etc.), which were usually utilized as effective methods to achieve high strength and sufficient ductility. The submicron eutectic Si could be achieved in Al-Si alloys by super-gravity solidification with a reliable elongation (over 10%) [14]. Nanoscale Si particles were observed in Al-7Si alloys by high-pressure solution treatment and aging with an excellent ductility (over 15%) [15]. Thus, the refinement of eutectic Si could significantly enhance the ductility of Al-Si alloys, even making it possible to undertake conventional plastic deformation, such as cold rolling.

Post plastic deformation (cold-rolling [16], hot-rolling [17], high pressure torsion [18], equal channel angular press, hot extrusion, forging [19]) was widely used to further enhance the strength in wrought Al alloys. Liao et al. [16] found that the hardness of Al-12Si-0.2 Mg alloy increased over 70% after cold rolling. Ma et al. [20] found that the enhanced toughness of Al-11Si alloys could be achieved by equal channel angular press, mainly due to the ultra-fine grain structure (~300 nm). Cepeda et al. [18] found that the enhanced hardness (~84 HV) of Al-7 wt.% Si alloy could be obtained after high pressure torsion, which was higher than that of as-cast alloy (~77 HV). Xu et al. [21] found that the improvement of plasticity and strength was detected after hot extrusion and T6 in Al-6Si-2Cu-0.5Mg alloy.

Based on the above discussion, the limited ductility could be detected in conventional Al-Si alloys mainly due to the needle-like eutectic Si. It is tough for this type of Al-Si alloy to further enhance the mechanical properties by the post plastic deformation. The refinement of eutectic Si in Al-Si alloy could be achieved by super-gravity solidification, resulting in the improvement of plasticity/ductility and strength, which may provide the possibility for this Al-Si alloy to suffer the conventional plastic deformation. Thus, it is our goal to evaluate whether the cold-rolling could be utilized to further improve the mechanical properties of Al-Si alloy. If it is possible, the cold rolling and annealing may affect the microstructure and mechanical properties of Al-Si alloy. It should be noted that the influence of cold rolling and annealing on Al-Si alloys solidified under a super-gravity field was not studied in the literature.

## 2. Experimental Processing

**Figure 1:**
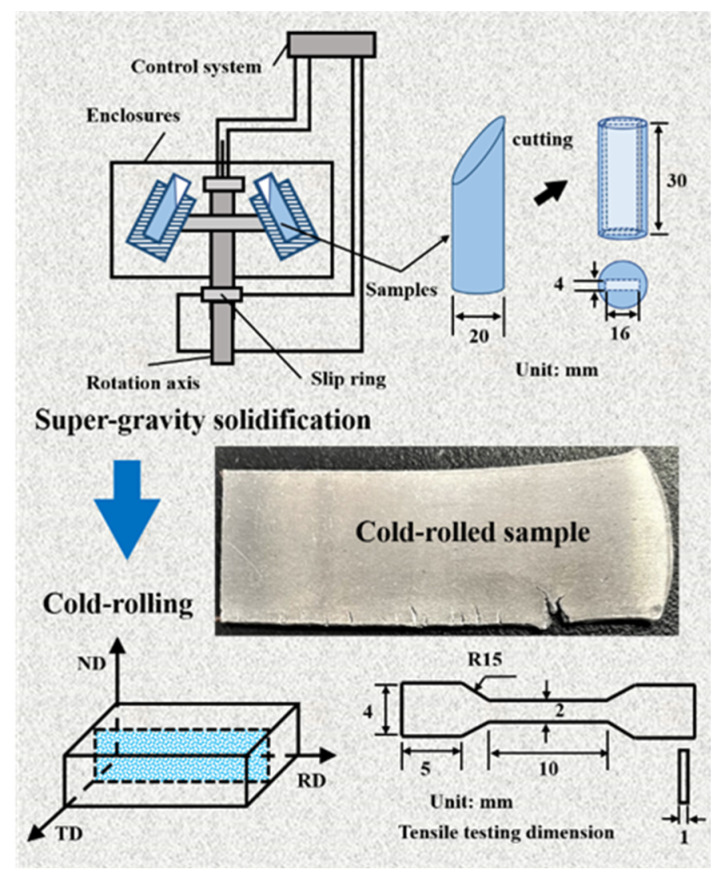
The flow process chart of super-gravity solidification and cold-rolling.

In this experiment, high-purity silicon (99.9%) and high-purity Al (99.9%) were used as raw materials. Al-14.5 wt.% Si alloy was put in a graphite crucible, then melted at 1123 K in a resistance furnace. After maintaining for 0.5~0.7 h, the melt was poured into a graphite tank to obtain an ingot. The ingot was cut into several pieces of ~50 g. The sample was then put into a cylindrical graphite crucible (φ20 mm) and re-melted at 1123 K. Subsequently, the melt was put into the centrifuge (TG1850-WS) and solidified under a super-gravity field (3000 g), which was designed as S0. A rectangular block (16 mm × 30 mm × 4 mm) was cut from the solidified sample, and cold-rolled with an overall reduction of 50% (~2 mm), which was designed as S1. The process flow chart of the experiment is provided in Figure 1. The cold-rolled samples were annealed at a temperature of 423 K. The annealed samples with various holding times (0.5 h, 1 h and 2 h) were, respectively, designated as S2, S3 and S4.

X-ray diffraction (XRD, Smartlab SE) was used to identify the phase of the samples with CuK_α_ (λ = 1.54051 nm) radiation. The measurement condition of all the samples was identical with a scan speed of 2°/min. An optical microscope (OM, Zeiss) was utilized to observe the etched sample (10 wt.% NaOH solution for 90 s). A scanning electron microscope (SEM, Nova 400) was used to observe the eutectic structure of each sample with the accelerating voltage of 20 KeV. The average length and width of the eutectic Si in various samples were measured from SEM images via Image J. The distribution of dislocation in the sample was characterized by transmission electron microscope (TEM, JEM-2100). The specimens for TEM analysis were prepared using an Ion Milling (Gatan PIPS 691). The Vickers hardness tester (HV-1000B) was used for testing the hardness of the samples with the loading force of 200 gf based on the standard of GB/T 4340.1-2009. The universal tensile testing machine (INSTRON 5900 Series) was used for testing the tensile properties of samples based on standard of GB/T 228.1-2010. The gauge length for each sample was ~10 mm, while the loading rate for the tensile testing was 0.01 mm/s. Three nominally identical tensile specimens were used for each test to obtain the average yield strength, tensile strength and elongation.

## 3. Results

### 3.1. X-ray Diffraction

**Figure 2:**
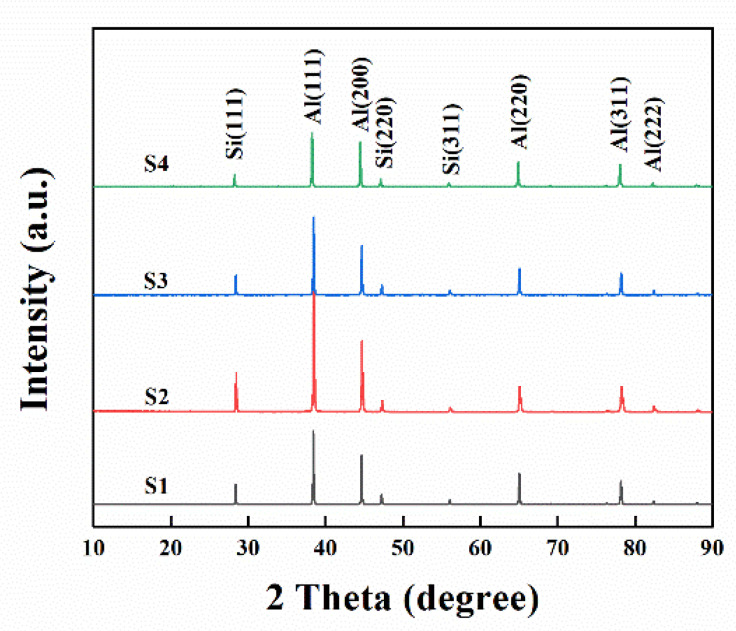
X-ray diffraction of as-rolled Al-Si alloys with various annealing time.

XRD patterns of all the samples are provided in Figure 2. The figure shows two phases of Al and Si, in which the main diffraction peaks of Al include (111), (200), (220) and (311), and the main diffraction peaks of Si include (111), (220) and (311). The strongest peaks of Al crystal and Si crystal remain unchanged in the (111) crystal plane. With the increase in annealing time, the diffraction peak intensity of Al increases significantly and reaches the peak at 0.5 h, while the diffraction peak of Si has the same trend. It should be noted that no new phase was detected in all the samples.

### 3.2. Microstructure Characterization

**Figure 3:**
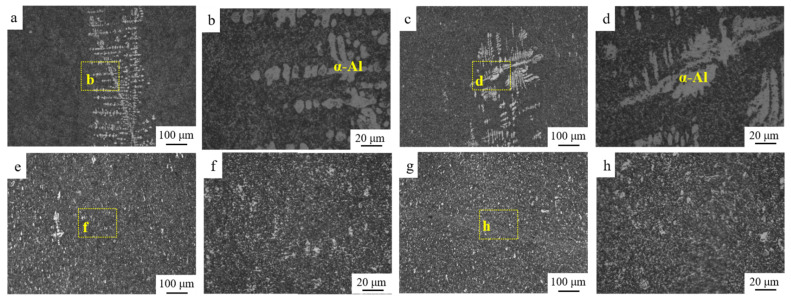
OM images showing the morphology and distribution of eutectic structure and α-Al in Al-Si alloys: (**a**,**b**) S0, (**c**,**d**) S1, (**e**,**f**) S3, and (**g**,**h**) S4.

**Figure 4:**
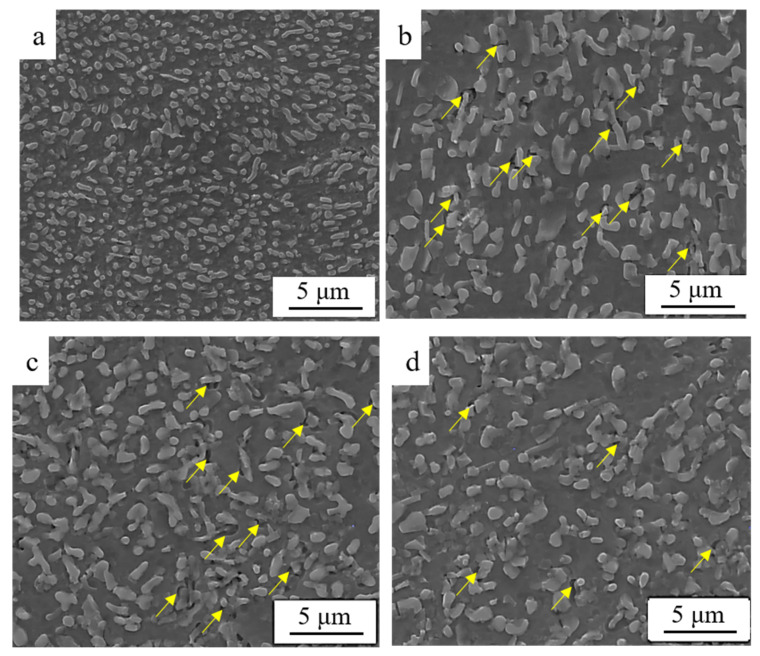
SEM images showing the morphology and distribution of eutectic Si in Al-Si alloys: (**a**) S0, (**b**) S1, (**c**) S3, and (**d**) S4.

The representative images showing the distribution of eutectic structure and α-Al in S0, S1, S3 and S4 are provided in Figure 3. Compared to S0 Figure 3b, the grain of α-Al in S1 Figure 3d was severely elongated along the rolling direction. The uniform structure was detected in S3 and S4 after annealing. The morphology and distribution of eutectic Si were provided in Figure 4. The size of the eutectic Si increases a bit after cold-rolling. The width and length of eutectic Si in all the samples are summarized in Table 1. It should be noted that several defects were observed in the interface between Al and eutectic Si, which were highlighted by yellow arrows in Figure 4. During annealing processing, a slight coarsening of eutectic Si was found in S3 and S4. In addition to the size, several eutectic Si with irregular granular morphology could be also detected in S3 and S4.

**Figure 5:**
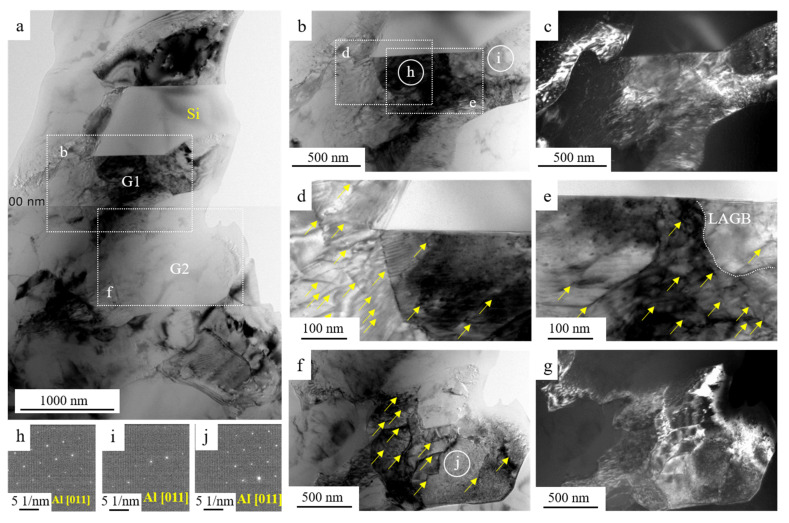
TEM images showing the distribution of dislocations in S1, (**a**) bright-field image showing a grain (G1) in the vicinity of Si particle while a grain (G2) away from Si, the inset image indicating the SAED of Si, (**b**,**c**) bright-field and dark-field images showing the distribution of dislocation in G1, (**d**,**e**) enlarged images in (**a**) showing the morphology of dislocation and LAGB around Si particle, (**f**,**g**) bright-field and dark-field images showing the distribution of dislocation in G2, (**h**–**j**) corresponding images showing the SAED in (**b**,**f**), respectively.

The representative TEM images of S1 are displayed in Figure 5. The eutectic Si with an angular morphology was detected in Figure 5a. This bright field image showed one Al grain in the vicinity of Si (G1) and one Al grain away from Si (G2). The G1 was tilted into the zone axis of [011]; a high density of dislocations was detected and could be observed in the vicinity of Si, as shown in the bright field image in Figure 5b and dark field image Figure 5c. The dislocation was marked by yellow arrows in the enlarged images, as provided in Figure 5d,e, The SAED patterns are provided in Figure 5h,i. The SAED in Figure 5h is in the zone axis of [011], while the SAED in Figure 5i is almost close to the zone axis of [011], indicating a low angle grain boundary (LAGB) in these two regions (marked by a white dotted line in Figure 5e. When tilting the G2 into the zone axis of [011], the different types of dislocations were visible, as provided in the bright field image Figure 5f and dark field image Figure 5g. The dislocation density in G2 was lower relative to that in G1.

**Figure 6:**
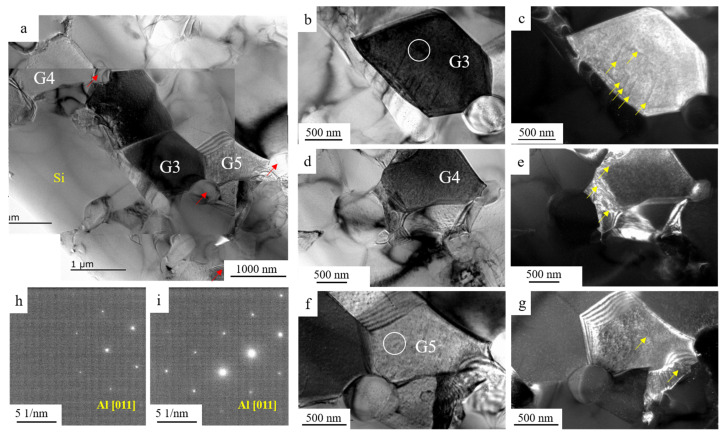
TEM images showing the distribution of dislocations in S4, (**a**) bright-field image showing two grains (G3 and G4) in the vicinity of Si particle while a grain (G5) away from Si, the inset image indicating the SAED of Si, (**b**,**c**) bright-field and dark-field images showing the distribution of dislocation in G3, (**d**,**e**) bright-field and dark-field images showing the distribution of dislocation in G4, and (**f**,**g**) bright-field and dark-field images showing the distribution of dislocation in G5, (**h**,**i**) corresponding images showing the SAED in (**b**,**f**), respectively.

The representative TEM images of S4 are provided in Figure 6. The size of Si in S4 was coarser than that in S1. G3 and G4 were located in the vicinity of Si, while G5 was located away from Si. These grains were tilted into the [011] zone axis to obtain the bright field and dark field images. The dislocation was marked by yellow arrows in Figure 6c,e,g. The dislocation density in S4 was significantly lower than that in S1. In addition to the dislocation, the recrystallized grains were detected in the triple grain boundary junction highlighted by red arrows in Figure 6a. The grain size of Al around eutectic Si in the S4 sample was finer relative to that in S1.

### 3.3. Mechanical Properties

**Figure 7:**
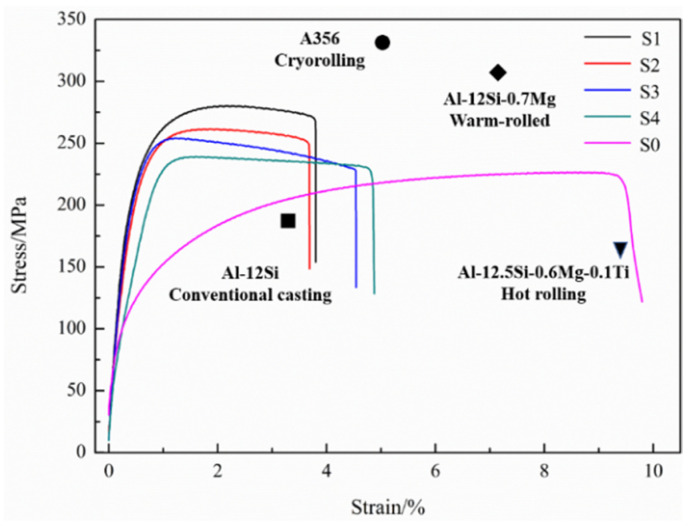
Tensile stress-strain curves of super-gravity solidified Al-Si alloys and as-rolled Al-Si alloys with various annealing time [17,22,23,24].

Figure 7 shows the tensile stress-strain curve of S0, S1, S2, S3 and S4. The yield strength (214 ± 11 MPa) of the cold-rolled S1 sample was significantly higher (~68.5%) than that of S0 (127 ± 8 MPa). However, limited elongation (~2.9 ± 0.5%) was detected after cold-rolling. The tensile strength and yield strength of the annealed sample gradually decrease with the increase in annealing time. The acceptable plasticity could be observed after 2 h annealing. The tensile strength in the current paper was higher compared with the commercial Al-12Si alloys [22], and Al-12.5Si-0.6Mg-0.1Ti alloys (hot-rolling) [17]. However, the tensile strength was lower than that of the cryo-rolled A356 alloys [23] and warm-rolled Al-12Si-0.7Mg alloys [24]. Compared with S0, the higher Vickers Hardness could be achieved after cold-rolling, however, a slight decrease was detected after annealing, as provided in Table 1.

### 3.4. Fractography

**Figure 8:**
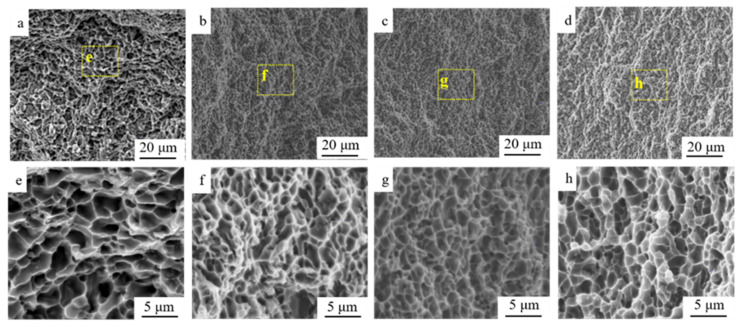
The fracture surface (after tensile testing) of Al-Si alloys: (**a**,**e**) S0, (**b**,**f**) S1, (**c**,**g**) S3, and (**d**,**h**) S4.

**Figure 9:**
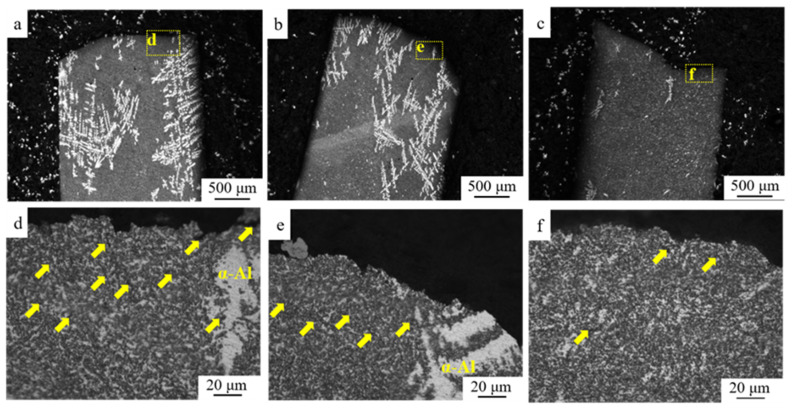
OM images showing the microstructure beneath the fracture surface of Al-Si alloys: (**a**,**d**) S1, (**b**,**e**) S2, and (**c**,**f**) S4.

The fracture surface after the tensile test of representative Al-Si alloys is provided in Figure 8. The typical ductile transgranular fracture was found on the fracture surface in S1; however, the lower depth of dimples with finer size in S1 was detected compared with that in S0. Generally, dimples were considered as the consequence of the initiation, growth and coarsening of micro-voids around the second particles [15,25]. The pile-up of dislocations in the Al-Si interface might lead to the formation of micro-voids. The morphology of the dimples was closely related to the time for the development of micro-voids. Typically, when the dimples were deeper and larger, the relatively higher elongation could be observed in the bulk [26]. The uniform and regular distribution of the dimples with a relatively larger size could be observed in S4 after annealing, indicating an increase in ductility/plasticity. The microstructure beneath the fracture surface of Al-Si alloys was obtained by OM images to show the initiation and propagation of cracks. The initiation of cracks preferred to occur in the vicinity of the inherent casting defects and the sharp edges of the Al-Si interface in Al-Si alloys [27]. Since the limited defects could be observed in the current Al-Si alloys solidified under a super-gravity field. Thus, the potential position for the initiation of cracks was at a weak Al-Si interface, then the cracks propagated through the matrix, as provided in Figure 9. The high density of micro-cracks (highlighted by yellow arrows) was detected in an as-rolled sample, as shown in Figure 9d (S1). After annealing, the number density of micro-cracks beneath the fracture surface decreased. Certain micro-cracks were found in the S2 sample while limited micro-cracks were detected in the S4 sample.

## 4. Discussion

### 4.1. Microstructure Evolution

#### 4.1.1. Microstructure Evolution during Cold-Rolling

The significant refining of the eutectic Si could be achieved by super-gravity solidification, making it possible for Al-Si alloys to undertake the post cold-rolling without catastrophic failure. Typically, the fragment of eutectic Si might occur during deformation and lead to the rearrangement of eutectic Si, thus the predominant changes in morphology and distribution of eutectic Si could be observed after deformation [28,29]. However, the sub-micron eutectic Si with homogeneous distribution could be obtained by solidification under a super-gravity field. Cold-rolling did not change the distribution and morphology of the eutectic Si, however, a slight increase in Si size and high density of dislocations were detected in the as-rolled sample (S1), as provided in Figure 5. In addition to the spatial distribution of eutectic Si, a high density of dislocation could be observed after cold-rolling, as provided in Figure 5. It should be noted that the dislocation density in the vicinity of the Si particle was higher than that far away from the Si particle. Thus, it is reasonable to assume that the movement of the dislocation was obstructed by submicron eutectic Si during deformation, leading to the accumulation of dislocation in the vicinity of eutectic Si particles. Similar phenomena were also reported previously [20]. The formation of lattice distortion may form during cold-rolling, leading to the thermodynamic imbalance and an increase in dislocations, which in turn caused high internal stress [16]. Previous results have confirmed that the Si particle may act as a barrier for dislocation movement during tensile strain and lead to the accumulation of dislocation [23]. Thus, the dislocation preferred to be pinned in the vicinity of Si particles. The relatively low ductility of the as-rolled sample could be attributed to the relatively low work hardening rates. The presence of high dislocation density could significantly affect the accumulation capacities of dislocation in as-rolled samples during tensile testing [30].

#### 4.1.2. Microstructure Evolution during Annealing

The coarsening of eutectic Si could be observed during annealing. The coarsening of eutectic Si was related to the diffusion and local concentration of Si atoms in Al. The diffusion rate of Si atoms in Al was closely related to the temperature. The extension of annealing time at 423 K might allow the diffusion of Si atoms to be reduced with a limited concentration gradient based on Fick’s second law [31,32]. In addition to the diffusion rate of Si, the local concentration of Si atoms may affect the growth of Si during annealing. The source of Si atoms mainly originated from the solute Si in Al. The solid solution of Si in Al showed a linear relationship with the temperature below the solidus curve, a higher solid solution could be detected with an increase in temperature. The solid solution of Si in Al at room temperature and 813 K were approximately 0.05 wt.% and 1.2 wt.%, respectively [29]. Thus, certain Si atoms might dissolve into the Al during an annealing temperature of 423 K, and the extension of annealing time may lead to the growth and coarsening of Si. Previous results have confirmed that the growth direction of eutectic Si in conventional Al-Si alloys was <110> or <100>, leading to the formation of rod-like and needle-like eutectic Si [33,34,35,36]. However, it is still unclear whether super-gravity solidification and cold-rolling affects the solid solution of Si in Al.

### 4.2. Fracture Behavior

In commercial Al-Si eutectic alloys, the crack/voids were usually detected around eutectic Si (needle-like, dozens of micron), leading to a limited ductility [37]. The refinement of eutectic Si could be observed by solidification under a super-gravity field. The enhancement of strength and ductility make it possible to suffer post cold-rolling. However, a relatively low ductility was detected for the cold-rolled sample. High dislocation density could be observed in the vicinity of Si, as discussed in the previous section. As provided in Figure 9d, the initiation of cracks prefers to occur around the eutectic Si particles during external tensile stress mainly due to the high stress concentration at the interface. The propagation of micro-cracks occurred along the Al. A high density of dislocations has already accumulated during cold-rolling, this type of dislocation could affect the movement of mobile dislocation as well as the accumulation capacity of dislocation. Although limited ductility was observed in the as-rolled sample, a high density of dimples, the typical feature for the plastic fracture, could be still detected on the fracture surface, as provided in Figure 8b, f. Thus, the decohesion and micro-crack in the interface between Al-Si were responsible for the initiation of micro-cracks, and finally, the propagation of multiple micro-cracks led to the catastrophic failure of the as-rolled Al-Si alloys during tensile testing.

The internal stress and lattice distortion were eliminated with an increase in annealing time. Compared with the S1 sample, the density of dislocation decreased, as provided in Figure 6. The accumulation capacity of dislocation in the annealing sample is higher than that of the as-rolled sample. The micro-crack preferred to initiate around Si particle and propagate along the Al matrix; the enhanced accumulation capacity of dislocation could contribute to the relatively higher ductility. In addition to the dislocation, the recrystallization occurred during annealing, eliminating the differences in the grain structure caused by deformation. The regular grain could be observed in Figure 6. Similar phenomena could be found in the as-rolled sample after heat treatment [24]. The final fracture of the annealed sample was mainly caused by the shear tearing of the ductile Al, as shown in Figure 8d,h. The relatively high ductility could be obtained with a sacrifice in strength for the sample after annealing.

## 5. Conclusions

In this paper, Al-14.5Si alloys were successfully obtained by super-gravity solidification and cold-rolling. The influence of annealing time on mechanical properties and microstructure of the Al-Si alloy were investigated. The conclusion could be summarized as follows:

1. The grain of α-Al in S1 was severely elongated along the rolling direction during cold-rolling. A slight coarsening of eutectic Si was found during cold-rolling and annealing. The dislocation density in the vicinity of Si was higher than that far away from Si in the S1 sample. Compared with the S1 sample, lower dislocation density was observed in the S4 sample.
2. Compared with the S0 sample (~127 ± 8 MPa), the higher yield strength (~214 ± 11 MPa) with limited elongation (~2.9 ± 0.5%) could be achieved after cold-rolling. After annealing for 1 h, the yield strength and elongation of the S3 sample were ~202 ± 8 MPa and ~3.7 ± 0.3%, respectively.
3. Multiple micro-cracks/voids were detected beneath the fracture surface with an increase in annealing time and the internal stress and lattice distortion were eliminated, leading to a decrease in micro-cracks/voids.

## Figures and Tables

**Table 1:** Tensile properties of rolled Al-14.5Si alloy solidified in a super-gravity field under different annealing times.

Sample	Annealing Time (h)	Width of Eutectic Si (μm)	Length of Eutectic Si (μm)	Yield Strength (MPa)	Tensile Strength (MPa)	Elongation (%)	Hardness (HV)
S0	/	0.30 ± 0.05	0.47 ± 0.06	127 ± 8	216 ± 12	8.4 ± 1.2	75 ± 3
S1	0	0.38 ± 0.03	0.68 ± 0.07	214 ± 11	280 ± 15	2.9 ± 0.5	79 ± 5
S2	0.5	0.42 ± 0.05	0.82 ± 0.06	202 ± 6	262 ± 11	2.8 ± 0.6	74 ± 2
S3	1	0.45 ± 0.03	0.85 ± 0.09	202 ± 8	250 ± 9	3.7 ± 0.3	78 ± 5
S4	2	0.50 ± 0.03	1.01 ± 0.12	162 ± 5	239 ± 11	3.9 ± 0.2	75 ± 4

## Data Availability

Data will be made available on request.
